# The role of asymmetric dimethylarginine (ADMA) in COVID-19: association with respiratory failure and predictive role for outcome

**DOI:** 10.1038/s41598-023-36954-z

**Published:** 2023-06-17

**Authors:** Emanuela Sozio, Juliane Hannemann, Martina Fabris, Adriana Cifù, Andrea Ripoli, Francesco Sbrana, Demetrio Cescutti, Luigi Vetrugno, Stefano Fapranzi, Flavio Bassi, Massimo Sponza, Francesco Curcio, Carlo Tascini, Rainer Böger

**Affiliations:** 1 Infectious Diseases Clinic, Azienda Sanitaria Universitaria del Friuli Centrale (ASUFC), Udine, Italy; 2 Institute of Clinical Pharmacology and Toxicology, University Medical Center Hamburg-Eppendorf, Hamburg, Germany; 3 Institute DECIPHER, German-Chilean Institute for Research on Pulmonary Hypoxia and Its Health Sequelae, Hamburg, Germany; 4 Istituto di Patologia Clinica, Azienda Sanitaria Universitaria Friuli Centrale - Udine (ASUFC), Udine, Italy; 5 Department of Medical Area (DAME), University of Udine, Udine, Italy; 6 Bioengineering Department, Fondazione Toscana Gabriele Monasterio, Pisa, Italy; 7 Lipoapheresis Unit - Reference Center for Diagnosis and Treatment of Inherited Dyslipidemias, Fondazione Toscana “Gabriele Monasterio”, Via Moruzzi 1, 56124 Pisa, Italy; 8 Department of Medical, Oral and Biotechnological Sciences, University of Chieti-Pescara, Chieti, Italy; 9 Emergency Radiology Department - Azienda Sanitaria, Universitaria del Friuli Centrale (ASUFC), Udine, Italia; 10 Department of Anesthesia and Intensive Care Medicine, Azienda Sanitaria Universitaria Friuli Centrale - Udine (ASUFC), Udine, Italy; 11 U.O. Malattie Infettive, Azienda Sanitaria Universitaria Integrata di Udine, Via Pozzuolo, 330, 33100 Udine, Italy

**Keywords:** Medical research, Biomarkers, Experimental models of disease

## Abstract

We aimed to assess the potential role of Asymmetric dimethylarginine (ADMA) in conditioning respiratory function and pulmonary vasoregulation during Severe Acute Respiratory Syndrome Coronavirus 2 (SARS-CoV2) infection. Within 72 h from admission, samples from 90 COVID-19 patients were assessed for ADMA, SDMA, L-arginine concentrations. In addition to classical statistics, patients were also clustered by a machine learning approach according to similar features. Multivariable analysis showed that C-reactive protein (OR 1.012), serum ADMA (OR 4.652), white blood cells (OR = 1.118) and SOFA (OR = 1.495) were significantly associated with negative outcomes. Machine learning-based clustering showed three distinct clusters: (1) patients with low severity not requiring invasive mechanical ventilation (IMV), (2) patients with moderate severity and respiratory failure whilst not requiring IMV, and (3) patients with highest severity requiring IMV. Serum ADMA concentration was significantly associated with disease severity and need for IMV although less pulmonary vasodilation was observed by CT scan. High serum levels of ADMA are indicative of high disease severity and requirement of mechanical ventilation. Serum ADMA at the time of hospital admission may therefore help to identify COVID-19 patients at high risk of deterioration and negative outcome.

## Introduction

Since February 2020, Europe found itself fighting against the severe acute respiratory syndrome coronavirus 2 (SARS-CoV2). Among the clinical parameters that turned out to be relevant for patient outcome, hypoxia was among the first guiding the approach both to understand and to revert the COVID-19 pathology^1^. Many patients with COVID-19 pneumonia demonstrate severe hypoxemia but do not appear to be in respiratory distress, configuring the strange phenomenon called “silent hypoxemia”^2,3^. Despite the variation in pulmonary pathophysiological observations made in the consecutive waves of the pandemic, this disconnection between gas exchange and lung mechanics in COVID-19 pneumonia appears as a hallmark of this infection; it is characterized by a significant dilation of the blood vessels and capillaries of the lung in a proportion of COVID-19 patients^4,5^.

The major endogenous mediator of vasodilation is nitric oxide (NO); it plays a central role in vascular homeostasis, since it also inhibits platelet adhesion and aggregation, monocyte adhesion and smooth muscle cell proliferation^6^. Nitric oxide synthase (NOS) enzymes catalyze the formation of NO from L-arginine and O_2_, leading to the collateral synthesis of L-citrulline. Production of NO is oxygen-dependent, but NO also regulates O_2_ delivery, locally through vasomotor control and centrally, through cardiovascular and respiratory responses. Asymmetric dimethylarginine (ADMA) is an endogenous competitive inhibitor of NOS that can replace L-arginine at its binding site, antagonizing endothelium-dependent vasodilation^7,8^. Therefore, the ratio of L-arginine (the substrate of endothelial NO synthase) and ADMA (the enzyme’s competitive inhibitor) has been suggested as a correlate of endothelial NO synthase substrate availability; like high ADMA concentration, low L-arginine/ADMA ratio has been shown to be associated with all-cause mortality rate^9^.

ADMA is increased in patients with pulmonary arterial hypertension and represents a risk marker for cardiovascular events and mortality in patients with cardiometabolic diseases, but also in the general population^9^. Elevation of circulating ADMA concentration is a common phenomenon observed in individuals exposed to chronic or chronic intermittent hypoxia, e.g., at high altitude^10,11^. By contrast, low concentrations of ADMA may be an adaptation mechanism to hypoxia by maintaining normal pulmonary blood flow. Indeed, there are reports suggesting that populations that are adapted to living at high altitudes are less affected by severe COVID-19^12^. In vitro experiments also demonstrate that hypoxia downregulates ACE2 and heparan sulfate expression in lung epithelial cells, thus reducing the binding ability of the spike protein of SARS-CoV2 and virus infectivity^13^. However, hypoxia is also a potent trigger of several molecular and cellular signals stimulating thrombogenesis^14^, an event that dominates COVID-19 endothelialitis^5,15^.

ADMA metabolism is complex. It is generated during physiological turnover of methylated proteins, together with monomethyl-L-arginine (NMMA) and symmetric dimethylarginine (SDMA)^16^. ADMA and NMMA are competitive inhibitors of NO synthesis, while SDMA does not directly inhibit NOS activity^17^. Furthermore, ADMA and SDMA interfere with the cellular uptake of L-arginine, potentially reducing the bioavailability of L-arginine as substrate for NOS activity^18^.

As it shows a central role in NO regulation during hypoxemia, we hypothesized that ADMA may be involved in the NO-dependent altered respiratory function and inappropriate regulation of vasodilation in COVID-19. To assess this hypothesis, we measured ADMA, SDMA, L-arginine and L-arginine/ADMA ratio in a series of moderately to severely diseased COVID-19 patients within 72 h of admission to hospital.

## Patients and methods

### Patients

This retrospective study was conducted on 90 COVID-19 patients (70% males; mean age 63 ± 12 years) admitted to Academic Hospital of Udine between July 2020 and March 2021 with a diagnosis of SARS-CoV2 infection as confirmed by a positive reverse transcriptase-polymerase chain reaction (RT-PCR) on nasopharyngeal swabs. Patients admitted to hospital due COVID-19 were eligible for inclusion. Exclusion criteria included pregnancy and being younger than 18 years of age. The spectrum of COVID-19 disease severity ranged from mild, self-limiting respiratory tract illness to severe progressive pneumonia, multi-organ failure, and death. Baseline characteristics included: age, gender, and blood exams noted to be related to disease severity and collected within 72 h of admission to the hospital such as white blood cell count (WBC), lymphocyte count, lactate dehydrogenase (LDH), Mid-Regional proadrenomedullin (MR-proADM), C-reactive Protein (CRP), Interleukin-6 (IL-6); a past medical history of cardiovascular disease, diabetes, chronic kidney disease, chronic respiratory diseases; Charlson Comorbidity Index (CCI); clinical severity with SOFA score and COVID-19 WHO Severity Classification^19^ upon hospital admission. All blood samples analyzed were collected as part of routine clinical care on admission to the hospital.

### Sample size analysis

The sample size was computed in relation to the analysis of variance for ADMA. Three groups were considered: patients without respiratory failure (Group 1), patients with respiratory failure and no IMV (Group 2), and patients with respiratory failure and IMV (Group 3). The effect size for ANOVA was determined considering the ADMA values reported in the literature^20^; the expected mean values of ADMA in the three groups were: 0.6, 0.6, 0.8 (µmol/L), while the SD was 0.2 (µmol/L), with an effect size of 0.471. A power of 80% and a type 1 error of 0.05 were then guaranteed by a sample of 48 patients (16 for each group). This number had to be corrected for the post-hoc comparisons. Our main interest in the difference between groups 2 and 3, with an expected effect size of 1, a power of 80% with a type 1 error of 0.017 (Bonferroni correction, 0.05/3) was guaranteed by a sample of 19 subjects for each group.

### Endpoints

The primary endpoint was the characterization of the parameters able to modulate hypoxic pulmonary vasoconstriction related to a negative outcome (respiratory failure and/or death) in patients admitted for COVID-19 disease. This assessment was done with a cluster analysis.

The explorative secondary endpoint was the investigation of all the factors influencing COVID-19 outcome evaluated as composite outcome of IMV and/or in-hospital death in COVID-19 disease.

### Study design and ethical approval

Patients were followed up on a daily basis until discharge or in-hospital death for clinical outcomes such as in-hospital mortality, need for invasive mechanical ventilation (IMV), need for transfer to Intensive Care Unit (ICU) or the presence of respiratory failure without need for IMV. Results from routine clinical laboratory assays were anonymously collected. Length of hospital stay for COVID-9 was calculated as the number of days between admission and discharge for surviving patients. Pulmonary vasodilatation was assessed with computed tomography (CT) performed on A 64 slice CT scanner (GE VCT Scanner; GE Medical Systems, Milwaukee, WI, USA). All CT scans were performed on patients in supine position, possibly in maximal inhalation. Acquisition parameters 120 kV 100-400mAs (18 Noise Index) pitch thickness 1.4 with standard acquisition and with kerner for the lung. Two radiologists with 10 and 30 years of thoracic imaging experience make a blinded evaluation of the pulmonary vasodilatation using ESTENSA software (Esaote S.p.a). Serum samples were collected within 72 h of admission to the hospital and stored in aliquots, immediately frozen at − 80 °C until analyzed for ADMA, SDMA, and L-arginine. Patients were enrolled in accordance with the Helsinki Declaration, the study was approved by the Udine University Hospital Institutional Review Board (Unique Protocol ID: Z7C2CA5837); and all methods were performed in accordance with the relevant guidelines and regulations. At hospital admission, patients were routinely asked for their consent to anonymized aggregate data analysis for research purposes through the General Electronic COnsents (GECO system). Additionally, we contacted survivors to confirm the permission to use their blood samples stored at -80 °C for the present study.

### Biochemical analyses

Analysis of L-arginine, ADMA, and SDMA in serum was assessed by ultra-performance liquid chromatography—tandem mass spectrometry (UPLC-MS/MS) using validated protocols established in our laboratory^21^. Briefly, 25 μl of serum were diluted in methanol to which stable isotope-labelled internal standards had been added. Subsequently, the compounds were converted into their butyl ester derivatives and quantified by UPLC-MS/MS (Xevo TQ-S cronos, Waters GmbH, Eschborn, Germany). Compounds were separated on an Aquity UPLC BEH C18 column (2.1 × 50 mm, 1.7 µm, Waters GmbH). The coefficient of variation for the quality control samples was below 15% for all compounds. ADMA, SDMA, and L-arginine were also analyzed in 38 blood donors who served as healthy controls.

MR-proADM plasma concentrations were measured in an automated Kryptor analyzer, using the TRACE technology (Kryptor; BRAHMS, Hennigsdorf, Germany). IL-6 serum concentrations were measured by microfluidic ultrasensitive ELISA using the Protein simple plex technology on ELLA instrument (R&D systems, Biotechne, USA). All other laboratory biomarkers were evaluated using routine certified diagnostic methods.

The manuscript was drafted according to the Standards for the Reporting of Diagnostic accuracy studies STARD criteria^22^.

### Statistical analyses

Variables are described as mean ± standard deviation for normally distributed variables, and as median and interquartile range or proportion for non-normally distributed variables. Accordingly, comparisons between patients were performed with a two-tailed unpaired t-test, Mann–Whitney test or chi-square test with continuity correction. Moreover, the AUCs of the SOFA and ADMA were calculated.

The relationship between covariates and the probability of IMV was assessed with logistic regression. For multivariable analysis, only covariates with *p* < 0.10 at univariable logistic regression were considered; given the small sample size and the number of covariates to be considered, a lasso penalised method was used for the multivariable logistic regression. Risk stratification was explored using an unsupervised machine learning approach^23^ from variables strictly related to the severity of disease (COVID-19 WHO Severity Classification, SOFA score, PaO_2_/FiO_2_), a random forest^24^ was built to discriminate between data structure and noise; the similarity between each couple of patients was computed as the percentage of trees in the forest that identically classify the couple; by a multidimensional scaling procedure, the similarity measure was transformed in a Euclidean distance and the patients represented as points in a plane; finally, by a Bayesian model-based approach^25^, distinct clusters (phenotypes) of patients were identified.

The machine learning identified phenotypes were characterized by one-way ANOVA analysis; Fisher test was used for normally distributed variables, Kruskal–Wallis test for non-normally distributed continuous variables, and Chi-square test for nominal variables; post-hoc comparisons were performed with Bonferroni correction. The relationship between ADMA, disease severity, and time from the onset of symptoms (≤ 10 days or > 10 days) was studied with a two-way ANOVA analysis. The R statistical software [R Core Team 2021] was used for all analyses, and a *p* value less than 0.05 was considered statistically significant. Negative outcome was assessed by the composite endpoint of IMV and/or death, due to low mortality rate.

### Ethics approval and consent to participate

Informed consent was obtained from all subjects and/or their legal guardian(s).

## Results

We enrolled 38 healthy subjects as controls for assessments of ADMA, SDMA, L-arginine. Serum concentrations in healthy subjects were 0.66 ± 0.10 µmol/L for ADMA, 0.36 ± 0.10 µmol/L for SDMA, and 136.1 ± 35.5 µmol/L for L-arginine.

### Baseline characteristics of the cohort of COVID-19 patients

Baseline characteristics of the 90 patients are summarized in Table 1.

**Table 1:** Clinical characteristics and laboratory findings of study patients.

	Overall (n 90)	Patients without respiratory failure (n 38)	Patients with respiratory failure who need IMV (n 22)	Patients with respiratory failure who required IMV (n 30)	*p*
Female	27 (30%)	13 (34%)	7 (32%)	7 (23%)	0.610
Age (years)	63 ± 12	62 ± 14	63 ± 11	65 ± 11	0.535
BMI	27.9 ± 3.9	28.5 ± 4.1	26.4 ± 3.2	29.1 ± 3.2	0.115
Cardiovascular disease	16 (18%)	7 (18%)	4 (18%)	5 (17%)	0.981
Diabetes	12 (13%)	3 (8%)	5 (23%)	4 (13%)	0.265
Charlson comorbidity index	2 [1–4]	3 [1–4]	2 [1–3]	3 [2, 3]	0.600
SOFA score	3 [1–3]	1 [1–3]°,§	2 ^2,3^*	3 [3–3]	< 0.001
COVID-19 WHO Severity Classification	3.7 ± 1.0	3.0 ± 0.8°,§	4.0 ± 0.4*	4.5 ± 0.4	< 0.001
Length of hospital stay (days)	11 ^7–19^	8 ^6–10^°,§	12 ^10–14^*	22 ^14–33^	< 0.001
Death	10 (11%)	0 (0%)^§^	0 (0%)*	10 (33%)	< 0.001
ICU admission	37 (41%)	1 (3%)°^,§^	6 (27%)*	30 (100%)	< 0.001
PaO_2_/FiO_2_ ratio	240 ± 109	314 ± 101°^,§^	175 ± 64	192 ± 89	< 0.001
White blood cell (/mmc)	6.62 ± 3.09	5.33 ± 2.16^§^	5.78 ± 2.28*	8.87 ± 3.23	< 0.001
Lymphocytes (/mmc)	0.72 [0.52–1.01]	0.76 [0.62–1.25]	0.84 [0.61–0.98]	0.63 [0.30–0.78]	0.084
LDH (U/L)	580 [440–812]	462 [390–579]°^,§^	626 [538–766]	813 [576–981]	< 0.001
C reactive protein (mg/dl)	58.7 [26.4–103.1]	41.2 [14.6–66.4] ^§^	52.2 [40.8–93]*	101.8 [58.5–161.1]	< 0.001
MR-proADM (nMol/L)	0.84 [0.68–1.25]	0.73 [0.61–1.03]^§^	0.82 [0.75–1.02]	1.21 [0.82–1.74]	< 0.001
IL-6 (pg/ml)	29.0 [12.4–64.8]	24.0 [6.3–63.5]	42.0 [22.0–72.3]	29.5 [15.5–73]	0.321
ADMA (µmol/L)	0.62 ± 0.20	0.55 ± 0.11^§^	0.52 ± 0.14*	0.79 ± 0.21	< 0.001
L-arginine (µmol/L)	146.56 ± 54.86	138.90 ± 61.51	156.60 ± 55.63	148.91 ± 44.46	0.469
L-arginine/ADMA	245.54 + / − 121.25	264.71 + / − 131.8	318.05 + / − 129.47	195.09 + / − 62.97^§^,*	< 0.001
SDMA (µmol/L)	0.57 [0.45–0.69]	0.57 [0.47–0.74]	0.50 [0.41–0.65]	0.58 [0.45–0.71]	0.213
Chest CT pulmonary vasodilation	51 (57%)	18 (47%) °	18 (82%)	15 (50%)	0.023

°*p* < 0.05 in Group 1 versus Group 2; ^§^*p* < 0.05 in Group 1 versus Group 3; *p < 0.05 in Group 2 versus Group 3.

During hospitalization, 38/90 patients did not develop respiratory failure, 22/90 patients developed respiratory failure but did not require IMV, while 30/90 patients required IMV. No significant differences in age, gender, and comorbidity were observed between these three patient groups. In-hospital mortality was 11% (10 out of 90 cases): all deceased patients had required IMV.

**Figure 1:**
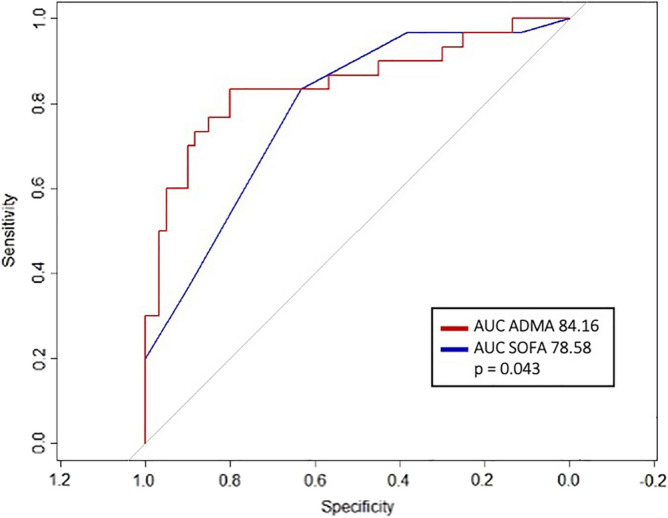
The AUCs of the SOFA and ADMA.

The AUCs of the SOFA score and serum ADMA levels compared to a negative outcome reveal a significantly higher AUC of ADMA with the respect to the SOFA one (see Fig. 1).

As shown in Table 1, upon post-hoc pairwise analysis, factors exhibiting a significant difference between all the 3 groups of patients were: SOFA score, COVID-19 WHO severity Classification, length of hospitalization (days), and need for ICU admission.

Patients who never developed respiratory failure during hospitalization showed a significantly higher PaO_2_/FiO_2_ ratio and significantly lower serum LDH activity as opposed to the other two groups.

Patients who required IMV during hospitalization showed significant differences compared to the other two groups in the following items: proportion of deceased patients (all belonging to the group of intubated patients), WBC, CRP, and L-arginine/ADMA ratio.

CT pulmonary vasodilation was significantly higher in patients with respiratory failure as opposed to patients not requiring IMV (82%), when compared to patients without respiratory failure (47%).

ADMA was significantly higher in patients with respiratory failure requiring IMV (0.79 ± 0.21) when compared to patients with respiratory failure without need of IMV (0.52 ± 0.14).

### Primary endpoint: the characterization of the parameters able to modulate hypoxic pulmonary vasoconstriction related to a negative outcome with a cluster analysis

The possible correlation between ADMA, SDMA, L-arginine values along with vasodilation at CT scan analysis (measured within 72 h from admission) and negative clinical outcome was studied using an unsupervised Random forest-based similarity map. This made it possible to identify 3 clusters of patients, which are explained below.

**Figure 2:**
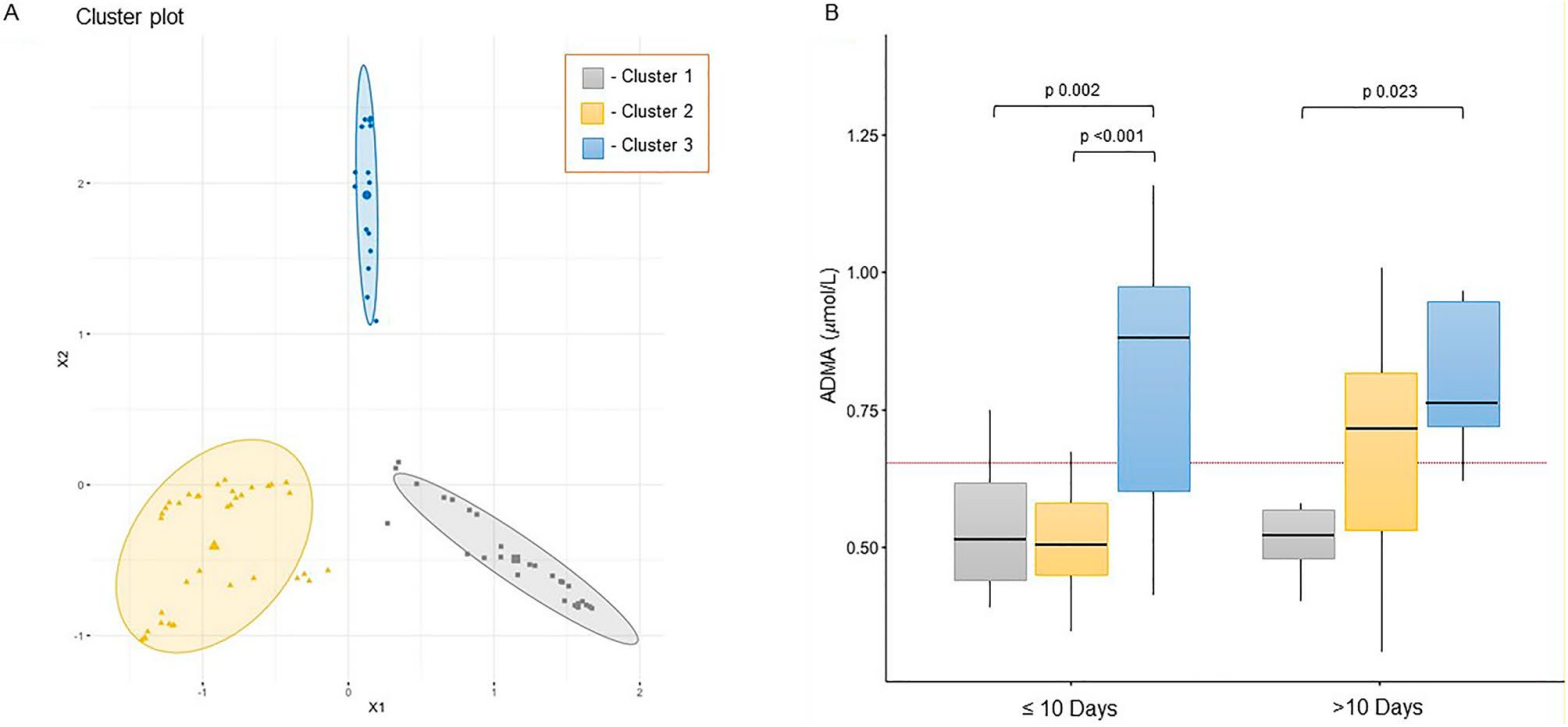
Panel (**A**) Random forest-based similarity map and clustering in three groups. Each point represents one patient; the higher the similarity (computed by unsupervised random forest) between each subject, the closer they are on the plot. Clustering was performed by a fuzzy c-means algorithm. Cluster 1 (n = 30; grey square) is characterized by a low group of disease severity, Cluster 2 (n = 43; yellow triangles) is characterized by an intermedia group of disease severity, Cluster 3 (n = 17; blue circles) is characterized by a high group of disease severity. Legend: X1 and X2 are the first two dimensions of the embedding space. Panel (**B**) Effect on the ADMA distribution with respect to the time from the onset of symptoms (*p* = 0.009) and the 3 Cluster of disease severity detected (*p* < 0.001).

Providing COVID-19 WHO Severity Classification, SOFA score, and PaO_2_/FiO_2_ ratio as severity-related variables, we tried to understand if there were clusters of patients with similar features in our cohort. A model-based clustering approach was used to identify distinct phenotypes (Fig. 2, Panel A): Cluster 1 (n = 30; grey squares) was characterized by low disease severity, Cluster 2 (n = 43; yellow triangles) was characterized by moderate disease severity, Cluster 3 (n = 17; blue circles) was characterized by high disease severity.

The characterization of the 3 Clusters is specifically summarized in Table 2.

**Table 2:** Characterization of the 3 clusters of patients identified by machine learning approach.

	Cluster 1 (n = 30)	Cluster 2 (n = 43)	Cluster 3 (n = 17)	*p*
Female	13 (43%)	7 (16%)°	7 (41%)	0.174
Age (years)	59 ± 14	67 ± 10°	62 ± 8	0.007
BMI	28.9 ± 4.4	26.4 ± 3.0	30.1 ± 3.7	0.935
Cardiovascular disease	2 (7%)	13 (30%)°	2 (12%)	0.038
Diabetes	3 (10%)	8 (19%)	2 (12%)	0.608
Charlson comorbidity index	1 [1–3]	3 [2–5]°	2 [1–3]*	< 0.001
SOFA score	1 [0.25–2]	3 [2, 3]°	3 [3, 4]^§,^*	< 0.001
COVID-19 WHO severity classification	2.6 ± 0.5	4.0 ± 0.0°	5.1 ± 0.2^§^,*	< 0.001
Length of hospital stay (days)	7 [6–9]	14 [10–20]°	20 [13–25]^§^	< 0.001
Death	0 (0%)	5 (12%) °	5 (29%)^§^	0.011
ICU admission	1 (3%)	19 (44%) °	17 (100%)^§,^*	< 0.001
IMV	1 (3%)	14 (33%) °	15 (88%)^§,^*	< 0.001
Patients without respiratory failure	27 (90%)	11 (24%)°	0 (0%)^§,^*	< 0.001
Patients with respiratory failure and without IMV	2 (7%)	18 (42%)°	2 (12%)*	< 0.001
PaO_2_/FiO_2_ ratio	342 ± 103	202 ± 69°	162 ± 65^§^	< 0.001
White blood cell (/mmc)	5.22 ± 2.00	6.51 ± 2.84	9.93 ± 3.46^§,^*	< 0.001
Lymphocytes (/mmc)	0.78 [0.69–1.27]	0.73 [0.42–0.97]	0.65 [0.56–0.79]	0.124
LDH (U/L)	480 [391–589]	619 [448–768] °	851 [688–1012] ^§^,*	< 0.001
C-reactive protein (mg/dl)	29.8 [11.5–75.4]	66.2 [41.2–105.3] °	89.4 [54.3–140.2] ^§^	0.005
MR-proADM (nMol/L)	0.70 [0.62–0.89]	0.97 [0.79–1.43] °	1.23 [0.82–1.69] ^§^	< 0.001
IL-6 (pg/ml)	21.0 [10.5–34.5]	50 [22–84] °	28 [14–42]	0.006
ADMA (µmol/L)	0.54 ± 0.12	0.6 ± 0.17	0.83 ± 0.24^§,^*	< 0.001
L-arginine (µmol/L)	151.76 ± 64.08	139.39 ± 52.76	152.13 ± 39.91	0.846
L-arginine/ADMA	291.38 ± 140.53	250.53 ± 118.74	187.92 ± 35.45 ^§^	0.005
SDMA (µmol/L)	0.54 [0.46–0.67]	0.54 [0.41–0.69]	0.61 [0.48–0.74]	0.736
Chest CT pulmonary vasodilation	14 (46%)	31 (72%)	6 (35%) *	0.014

°*p* < 0.05 in Group 1 versus Group 2; ^§^*p* < 0.05 in Group 1 versus Group 3; *p < 0.05 in Group 2 versus Group 3.

According to severity cluster analysis, post-hoc pairwise analysis identified the following factors exhibiting significant differences between the 3 groups of patients, especially when cluster 3 was compared to clusters 1 and 2 (see Table 2): need for ICU admission, patients who required IMV, LDH levels and ADMA levels at admission. In particular, ADMA levels were significantly higher in patients from Cluster 3 (0.83 ± 0.24 µmol/L) compared to both Cluster 1 (0.54 ± 0.12 µmol/L) and 2 (0.6 ± 0.17 µmol/L). Of note, cluster 2 was also characterized by a greater number of patients who did not require intubation as opposed to patients within Cluster 3, whilst still presenting with high proportions of respiratory failure (*p* < 0.001) as well as a high level of vasodilation (72%, according to CT scan (72% in cluster 2 vs 35% in cluster 3, *p* = 0.014).

The relationship between ADMA levels, disease severity (identified by clustering), and time from symptoms onset was assessed. Results are displayed in Fig. 2—Panel B. For patients admitted to the hospital within 10 days from onset of symptoms, ADMA levels were significantly higher in Cluster 3 as opposed to Clusters 1 and 2. The latter observation no longer persisted when ADMA levels were assessed in patients hospitalized after 10 days from symptoms onset. Indeed, 4 patients died within the Cluster 2 group with symptom onset beyond 10 days from admission (21 patients overall). ADMA levels in these 4 patients were elevated, ranging from 0.71 to 1 µmol/L, all of which were above the median serum ADMA level of cluster 2 (0.6 µmol/L).

### Secondary endpoint: analysis of factors influencing COVID-19 outcome

In order to better explore the outcome related to mortality, comparisons between survivors and deceased patients were performed (see Supplementary Table 1).

As shown in Supplementary Table 1, the deceased patients had a higher SOFA score and higher class of COVID-19 WHO Severity Classification as opposed to survivors; they required ICU admission and IMV. All deceased patients had lower PaO_2_/FIO_2_ ratio, higher LDH upon admission, higher ADMA levels, and lower L-arginine/ADMA ratio.

A sub-analysis of the 30 patients requiring IMV was carried out, comparing the survivors with the deceased (see Supplementary Table 2): no statistically significant differences were found.

Since all deceased patients required ICU admission and IMV, we performed a multivariable penalisted logistic regression analysis to investigate the association with the composite outcome of IMV and/or death (Table 3).

**Table 3:** Multivariable penalised logistic regression analysis performed to investigate the association with the composite outcome of IMV and/or death in COVID-19 patients.

	Univariable logistic regression			Multivariable penalized logistic regression °
	OR	95% CI OR	*p*	OR
Female	0.56	0.196–1.467	0.253	
Age	1.024	0.986–1.067	0.224	
BMI	1.089	0.923–1.288	0.307	
Cardiovascular disease	1.028	0.323–3.029	0.961	
Diabetes	1.204	0.336–3.971	0.764	
Charlson comorbidity index	1.064	0.848–1.334	0.585	
SOFA score	2.837	1.814–4.951	< 0.001	1.495
COVID-19 WHO severity classification	11.941	4.365–50.062	< 0.001	–
PaO_2_/FiO_2_ ratio	0.993	0.987–0.997	0.006	–
White blood cell	1.578	1.305–1.991	< 0.001	1.118
Lymphocytes	0.39	0.119–1.128	0.097	
LDH	1.004	1.002–1.006	< 0.001	–
C-reactive protein	1.017	1.009–1.027	< 0.001	1.012
MR-proADM	4.08	1.735–11.334	0.003	–
IL-6	1.005	1.001–1.011	0.04	–
ADMA *	2.563	1.801–3.953	< 0.001	4.652
L-arginine	1.001	0.992–1.008	0.924	
L-arginine/ADMA	0.99	0.982–0.995	0.002	–
SDMA	1.081	0.299–3.427	0.893	
Chest CT pulmonary vasodilation	0.385	0.147–0.986	0.048	–

*OR calculated for variation of 0.1 µmol/L in ADMA value; °Multivariable penalised logistic regression analysis furnishes the OR of the selected covariates, without confidence intervals.

The multivariable penalisted logistic regression analysis showed that two factors were significantly associated with negative outcome: SOFA score (OR 1.495), White blood cell (OR 1.118), C-reactive protein (OR 1.012) and serum ADMA levels (OR 4.652).

## Discussion

The present study provides evidence that patients hospitalized with COVID 19 disease cluster into three groups with distinct symptoms, disease severity, and prognosis. Specifically, patients displaying signs of respiratory failure may or may not need mechanical ventilation; the latter group is characterized by pulmonary vasodilation and low ADMA concentration. Instead, ADMA levels are significantly higher in patients requiring mechanical ventilation.

The COVID-19 infection has been described to have three consecutive phases: early infection, pulmonary involvement, and severe hyperinflammation^26^. During the early phase of infection, the virus infiltrates the pulmonary parenchyma and starts replicating, causing an inflammatory response involving local vasodilation and increased endothelial permeability^27^. Early stages of COVID-19 may be associated with silent hypoxia and poor oxygenation. These paradoxical findings may be explained by impaired hypoxic pulmonary vasoconstriction in the infected lung and hypoxemia may best be explained by the loss of regulation of perfusion, i.e. by loss of hypoxic vasoconstriction^28,29^.

Our data suggest that this phenomenon might be related to low levels of ADMA; it may explain the increased pulmonary shunts and mismatch of ventilation/perfusion, observed in cluster 2 of this study.

ADMA and SDMA production occur by enzymatic degradation of protein during the response to hypoxia; they are involved in modulating cardiovascular function and the innate immune response by decreasing cellular L-arginine uptake^17,30^. Organ failure, cardiovascular thromboembolism, and kidney and liver failure may be causes and consequences of the imbalance of these biomarkers in COVID-19 disease.

The multivariable penalized logistic regression analysis, carried out to investigate the association with the composite outcome of IMV and/or in-hospital death, confirms that ADMA levels at admission have a prognostic role. Hannemann and co-workers previously still demonstrated that high levels of both, ADMA and SDMA, are linked to COVID-19 disease severity^20^ inpatients from the first wave. This present study comprised also patients during the second wave and the number of patients is higher than in the previous publication.

Previous studies of ADMA in sepsis and during infections in general have given conflicting results. In vitro^31^ and in vivo^32,33^ studies (in septic patients) suggest that inflammation and especially IL-6 have the ability to induce the activity of the DDAH enzyme with the result of lowering ADMA levels. By contrast, Xiao and colleagues^34^ demonstrated downregulation of DDAH2 and upregulation of protein arginine methyltransferase-1 (PRMT1; the enzyme that catalyzes the biosynthesis of ADMA) by LPS in rats. Böger^35^ proposed that downregulation or inactivation of DDAH may be counter-regulatory mechanisms in sepsis, limiting NO production by inducible NO synthase through elevated ADMA. In line with this, Zoccali and co-workers^33^ showed that ADMA plasma levels increase during the resolution of infection/inflammation. Iapichino et al.^32^ showed that in patients admitted to ICU for sepsis, the levels of ADMA and the ADMA/SDMA ratio were inversely proportional to the levels of inflammatory markers (CRP and IL-6). Conversely, Ghashut and colleagues reported elevated ADMA concentration at admission to be associated with mortality in a large study of 104 patients with critical illness^36^. Winkler et al. recently observed that increased plasma concentrations of SDMA and ADMA are associated with sepsis severity^37^. A meta-analysis of six prospective clinical studies including 705 patients with critical illness confirmed the association between high ADMA plasma concentration at admission and mortality (pooled odds ratio 3.13 (95% CI 1.78–5.51)^38^.

The lung is a major source of ADMA; several studies revealed that the complete pathway of ADMA biosynthesis by PRMTs and degradation by DDAHs is present in the lungs^16,39^. In an animal model of prolonged critical illness, DDAH activity was the main regulator of tissue and plasma ADMA concentrations^40^. This is in line with reports from DDAH1 knockout mice that are characterized by increased ADMA, impaired vascular NO release, endothelial dysfunction, and systemic and pulmonary arterial hypertension^41–43^. In the lungs, experimental overexpression of DDAH2 attenuated LPS-induced vascular leak in acute lung injury^44^. Hannemann and Böger recently provided evidence suggesting that regulation of DDAH1 (down) and DDAH2 (up) in the lungs during hypoxia is involved in modulating hypoxic pulmonary vasoconstriction^45^. In an elegant study using isoform-specific siRNAs, Wang and co-workers showed that expressional repression of DDAH1 increased circulating ADMA concentration but did not have a major impact on vascular function, whilst expressional repression of DDAH2 caused vascular dysfunction whilst leaving plasma ADMA concentration largely unchanged^46^. Therefore, whilst the direct role of DDAH2 in enzymatic cleavage of ADMA has remained controversial, both isoforms seem to play distinct roles in regulating NO-mediated vascular function.

These observations may help to explain the relationship between ADMA and pulmonary vasodilation observed in COVID-19 patients with respiratory impairment in the present study. SARS-CoV-2, by binding to ACE2 receptor for host cell entry^47,48^, has a strong organotropy towards the lung circulation, causing endothelitis and pulmonary vascular dysregulation^5,15^. Consequences arising from this for the integrity of the pulmonary L-arginine/ADMA/NO pathway may result in differences in pulmonary vascular response to SARS-CoV-2 infection, and determine the clinical course and outcome of patients.

The three clusters identified by means of the unsupervised machine learning approach clearly reflect the clinically identified subgroups of COVID-19 patients. Cluster 1 was mainly composed of patients who did not develop respiratory failure; Clusters 2 and 3 comprised patients who developed respiratory failure; amongst these, patients in cluster 2 had no clinical need for IMV, low ADMA concentration at admission, and pulmonary vasodilation in CT (72% of patients in this cluster), whilst patients in cluster 3 included almost all patients requiring IMV, showed significantly less pulmonary vasodilation (35% of patients), had the most critical clinical course, and experienced the highest mortality rate.

A difference in vasodilation status was also observed among the clusters, notably, Cluster 2 showed greater vasodilation according to CT scan analysis as opposed to cluster 3.

An additional observation in this study was that ADMA levels upon hospitalization for COVID-19 disease may depend on the time elapsed from symptoms onset. For patients admitted to hospital within 10 days, ADMA levels were significantly higher in cluster 3 than in cluster 2. By contrast, no differences in ADMA levels were observed between clusters 2 and 3 at the time of hospitalization if more than 10 days had elapsed from symptoms onset. This may point to the temporal pattern of regulation of the L-arginine/ADMA/NO pathway during SARS-CoV-2 infection. However, cluster 2 also comprised four patients displaying exceptionally high levels of ADMA who had been admitted later than 10 days after symptom onset, and all of whom died during hospital treatment. Future studies may reveal whether and how the temporal associations of ADMA plasma concentration, pulmonary vasodilation, and the patient’s ability to overcome the pathophysiological consequences of SARS-CoV-2 infection may determine clinical course and outcome.

With the introduction of a measure of pulmonary vasodilation, this study comes one step closer to the suggested pathomechanism of ADMA effects in COVID-19 pneumonia.

Our study has strengths and limitations. We included 90 consecutive patients with moderate to severe COVID-19 disease requiring in-hospital treatment, and we rigorously subjected patients to pulmonary CT scan, biomarker measurements by the analytical gold standard, LC–MS/MS, machine learning-based cluster analysis, and follow-up for in-hospital course and outcome. Nonetheless, this was a retrospective single-center study, and the limited number of patients curtailed our ability to perform more subgroup analyses. The comparatively small sample size of COVID-19 patients admitted to ICU may also be a cause that we did not find differences in SDMA plasma concentration between the three subgroups of COVID-19 patients, which is in discrepancy to a previous study by Hannemann et al.^20^ that comprised a small group of patients with a broader range of disease severity. Furthermore, follow-up was limited by the duration of in-hospital treatment; we have no information on outcome after discharge. Finally, the molecular mechanisms underlying the regulation of ADMA in COVID-19 patients could not be revealed in this clinical study; animal or in vitro studies will be needed to shed light on this.

Attempts to improve endothelial dysfunction in COVID-19 patients have been made. In line with the available literature, our study supports observations that COVID-19 is featured by an endothelial dysfunction that may be causally related to dysregulation of the L-arginine/ADMA NO pathway. Therapeutic approaches such as inhaled NO administration in order to restore NO function may provide a valid strategy in order to treat COVID-19 disease; however, pilot studies have so far produced discrepant results^49–51^. Similarly, a pilot trial using sildenafil to improve pulmonary ventilation/perfusion mismatch generated unclear benefit^52^. In our study, low Larginine/ADMA ratio was associated to worse outcome. Thus, intervention on endothelial dysfunction, e.g., by L-arginine supplementation, may reduce the need for respiratory support as well as the length of hospital stay. Indeed, in an interim analysis of a randomized prospective trial conducted by Fiorentino and co-workers, L-arginine supplementation was shown to reduce oxygen requirement of COVID-19 patients^53^.

In conclusion, our study that distinct subtypes of COVID-19 patients can be identified that have different clinical disease severity and outcome. The combination of established clinical information with ADMA as a prognostic biomarker allows early identification of these subgroups. Machine learning approaches can help to improve the clustering of patients and clinical decision-making.

## Supplementary Information

Supplementary Tables.

## Data Availability

The datasets used and/or analyzed during the current study are available from the corresponding author on reasonable request.

## References

[1] Gattinoni L, Gattarello S, Steinberg I, Busana M, Palermo P, Lazzari S, Romitti F, Quintel M, Meissner K, Marini JJ, Chiumello D, Camporota L (2021). COVID-19 pneumonia: Pathophysiology and management. Eur. Respir. Rev..

[2] Busana M, Gasperetti A, Giosa L, Forleo GB, Schiavone M, Mitacchione G, Bonino C, Villa P, Galli M, Tondo C, Saguner A, Steiger P, Curnis A, Dello Russo A, Pugliese F, Mancone M, Marini JJ, Gattinoni L (2021). Prevalence and outcome of silent hypoxemia in COVID-19. Minerva Anestesiol..

[3] Simonson TS, Baker TL, Banzett RB, Bishop T, Dempsey JA, Feldman JL, Guyenet PG, Hodson EJ, Mitchell GS, Moya EA, Nokes BT, Orr JE, Owens RL, Poulin M, Rawling JM, Schmickl CN, Watters JJ, Younes M, Malhotra A (2021). Silent hypoxaemia in COVID-19 patients. J. Physiol..

[4] Lang M, Som A, Mendoza DP, Flores EJ, Reid N, Carey D, Li MD, Witkin A, Rodriguez-Lopez JM, Shepard JO, Little BP (2020). Hypoxaemia related to COVID-19: Vascular and perfusion abnormalities on dual-energy CT. Lancet Infect. Dis..

[5] Ackermann M, Verleden SE, Kuehnel M, Haverich A, Welte T, Laenger F, Vanstapel A, Werlein C, Stark H, Tzankov A, Li WW, Li VW, Mentzer SJ, Jonigk D (2020). Pulmonary vascular endothelialitis, thrombosis, and angiogenesis in Covid-19. N. Engl. J. Med..

[6] Böger RH, Bode-Böger SM, Frölich JC (1996). The L-arginine-nitric oxide pathway: Role in atherosclerosis and therapeutic implications. Atherosclerosis.

[7] Böger RH, Bode-Böger SM, Szuba A, Tsao PS, Chan JR, Tangphao O, Blaschke TF, Cooke JP (1998). Asymmetric dimethylarginine (ADMA): A novel risk factor for endothelial dysfunction: Its role in hypercholesterolemia. Circulation.

[8] Böger RH (2006). Asymmetric dimethylarginine (ADMA): A novel risk marker in cardiovascular medicine and beyond. Ann. Med..

[9] Böger RH, Sullivan LM, Schwedhelm E, Wang TJ, Maas R, Benjamin EJ, Schulze F, Xanthakis V, Benndorf RA, Vasan RS (2009). Plasma asymmetric dimethylarginine and incidence of cardiovascular disease and death in the community. Circulation.

[10] Lüneburg N, Siques P, Brito J, De La Cruz JJ, León-Velarde F, Hannemann J, Ibanez C, Böger RH (2017). Long-term intermittent exposure to high altitude elevates asymmetric dimethylarginine in first exposed young adults. High Alt. Med. Biol..

[11] Siques P, Brito J, Schwedhelm E, Pena E, León-Velarde F, De La Cruz JJ, Böger RH, Hannemann J (2019). Asymmetric dimethylarginine at sea level is a predictive marker of hypoxic pulmonary arterial hypertension at high altitude. Front. Physiol..

[12] Arias-Reyes C, Zubieta-DeUrioste N, Poma-Machicao L, Aliaga-Raduan F, Carvajal-Rodriguez F, Dutschmann M, Schneider-Gasser EM, Zubieta-Calleja G, Soliz J (2020). Does the pathogenesis of SARS-CoV-2 virus decrease at high-altitude?. Respir. Physiol. Neurobiol..

[13] Prieto-Fernández E, Egia-Mendikute L, Vila-Vecilla L, Bosch A, Barreira-Manrique A, Lee SY, García-Del Río A, Antoñana-Vildosola A, Jiménez-Lasheras B, Moreno-Cugnon L, Jiménez-Barbero J, Berra E, Ereño-Orbea J, Palazon A (2021). Hypoxia reduces cell attachment of SARS-CoV-2 spike protein by modulating the expression of ACE2, neuropilin-1, syndecan-1 and cellular heparan sulfate. Emerg. Microbes Infect..

[14] Gupta N, Zhao YY, Evans CE (2019). The stimulation of thrombosis by hypoxia. Thromb Res..

[15] Mosleh W, Chen K, Pfau SE, Vashist A (2020). Endotheliitis and endothelial dysfunction in patients with COVID-19: Its role in thrombosis and adverse outcomes. J Clin Med..

[16] Böger R, Hannemann J (2020). Dual role of the L-arginine-ADMA-NO pathway in systemic hypoxic vasodilation and pulmonary hypoxic vasoconstriction. Pulm Circ..

[17] Hannemann J, Zummack J, Hillig J, Böger R (2020). Metabolism of asymmetric dimethylarginine in hypoxia: From bench to bedside. Pulm Circ..

[18] Closs EI, Basha FZ, Habermeier A, Förstermann U (1997). Interference of L-arginine analogues with L-arginine transport mediated by the y+ carrier hCAT-2B. Nitric Oxide.

[19] World Health Organization. Clinical management of COVID-19: Interim guidance, 27 May 2020. World Health Organization. https://apps.who.int/iris/handle/10665/332196. License: CC BY-NC-SA 3.0 IGO (2020).

[20] Hannemann J, Balfanz P, Schwedhelm E, Hartmann B, Ule J, Müller-Wieland D, Dahl E, Dreher M, Marx N, Böger R (2021). Elevated serum SDMA and ADMA at hospital admission predict in-hospital mortality of COVID-19 patients. Sci Rep..

[21] Schwedhelm E, Maas R, Tan-Andresen J, Schulze F, Riederer U, Böger RH (2007). High-throughput liquid chromatographic-tandem mass spectrometric determination of arginine and dimethylated arginine derivatives in human and mouse plasma. J. Chromatogr. B Anal. Technol. Biomed. Life Sci..

[22] Bossuyt PM, Reitsma JB, Bruns DE, Gatsonis CA, Glasziou PP, Irwig L, Lijmer JG, Moher D, Rennie D, de Vet HC, Kressel HY, Rifai N, Golub RM, Altman DG, Hooft L, Korevaar DA, Cohen JF, STARD Group (2015). STARD 2015: An updated list of essential items for reporting diagnostic accuracy studies. Clin. Chem..

[23] Shi T, Horvath S (2006). Unsupervised learning with random forest predictors. J. Comput. Graph Stat..

[24] Breiman L (2001). Random forest. Mach. Learn..

[25] Fraley C, Raftery A (2002). Model-based clustering, discriminant analysis and density estimation. JASA.

[26] Pfeifer M, Ewig S, Voshaar T, Randerath WJ, Bauer T, Geiseler J, Dellweg D, Westhoff M, Windisch W, Schönhofer B, Kluge S, Lepper PM (2020). Position paper for the state-of-the-art application of respiratory support in patients with COVID-19. Respiration.

[27] Peiris JS, Lai ST, Poon LL, Guan Y, Yam LY, Lim W, Nicholls J, Yee WK, Yan WW, Cheung MT, Cheng VC, Chan KH, Tsang DN, Yung RW, Ng TK, Yuen KY, SARS study group (2003). Coronavirus as a possible cause of severe acute respiratory syndrome. Lancet.

[28] Gattinoni L, Chiumello D, Rossi S (2020). COVID-19 pneumonia: ARDS or not?. Crit. Care..

[29] Gattinoni L, Chiumello D, Caironi P, Busana M, Romitti F, Brazzi L, Camporota L (2020). COVID-19 pneumonia: Different respiratory treatments for different phenotypes?. Intensive Care Med..

[30] Tannheimer M, Hornung K, Gasche M, Kuehlmuss B, Mueller M, Welsch H, Landgraf K, Guger C, Schmidt R, Steinacker JM (2012). Decrease of asymmetric dimethylarginine predicts acute mountain sickness. J. Travel Med..

[31] Ueda S, Kato S, Matsuoka H, Kimoto M, Okuda S, Morimatsu M, Imaizumi T (2003). Regulation of cytokine-induced nitric oxide synthesis by asymmetric dimethylarginine: Role of dimethylarginine dimethylaminohydrolase. Circ. Res..

[32] Iapichino G, Umbrello M, Albicini M, Spanu P, Bellani G, Polli F, Pavlovic R, Cugno M, Fermo I, Paroni R (2010). Time course of endogenous nitric oxide inhibitors in severe sepsis in humans. Minerva Anestesiol..

[33] Zoccali C, Maas R, Cutrupi S, Pizzini P, Finocchiaro P, Cambareri F, Panuccio V, Martorano C, Schulze F, Enia G, Tripepi G, Boger R (2007). Asymmetric dimethyl-arginine (ADMA) response to inflammation in acute infections. Nephrol. Dial. Transpl..

[34] Xiao HB, Sui GG, Lu XY, Sun ZL (2018). Elevated Levels of ADMA Are Associated with Lower DDAH2 and Higher PRMT1 in LPS-Induced Endometritis Rats. Inflammation.

[35] Böger RH (2006). Live and let die: Asymmetric dimethylarginine and septic shock. Crit. Care..

[36] Ghashut RA, Blackwell S, Ryan S, Willox L, McMillan DC, Kinsella J, Talwar D (2017). Assessment of asymmetrical dimethylarginine metabolism in patients with critical illness. Eur J Clin Invest..

[37] Winkler MS, Nierhaus A, Rösler G, Lezius S, Harlandt O, Schwedhelm E, Böger RH, Kluge S (2018). Symmetrical (SDMA) and asymmetrical dimethylarginine (ADMA) in sepsis: High plasma levels as combined risk markers for sepsis survival. Crit Care..

[38] Mortensen KM, Itenov TS, Hansen MB, Hvid K, Lundstrøm LH, Bestle MH (2019). Mortality in critical illness: The impact of asymmetric dimethylarginine on survival-A systematic review and meta-analysis. Acta Anaesthesiol Scand..

[39] Bulau P, Zakrzewicz D, Kitowska K, Leiper J, Gunther A, Grimminger F, Eickelberg O (2007). Analysis of methylarginine metabolism in the cardiovascular system identifies the lung as a major source of ADMA. Am. J. Physiol. Lung. Cell. Mol. Physiol..

[40] Davids M, Richir MC, Visser M, Ellger B, van den Berghe G, van Leeuwen PA, Teerlink T (2012). Role of dimethylarginine dimethylaminohydrolase activity in regulation of tissue and plasma concentrations of asymmetric dimethylarginine in an animal model of prolonged critical illness. Metabolism.

[41] Leiper J, Nandi M, Torondel B, Murray-Rust J, Malaki M, O'Hara B, Rossiter S, Anthony S, Madhani M, Selwood D, Smith C, Wojciak-Stothard B, Rudiger A, Stidwill R, McDonald NQ, Vallance P (2007). Disruption of methylarginine metabolism impairs vascular homeostasis. Nat. Med..

[42] Hu X, Xu X, Zhu G, Atzler D, Kimoto M, Chen J, Schwedhelm E, Lüneburg N, Böger RH, Zhang P, Chen Y (2009). Vascular endothelial-specific dimethylarginine dimethylaminohydrolase-1-deficient mice reveal that vascular endothelium plays an important role in removing asymmetric dimethylarginine. Circulation.

[43] Hannemann J, Glatzel A, Hillig J, Zummack J, Schumacher U, Lüneburg N, Harbaum L, Böger R (2020). Upregulation of DDAH2 limits pulmonary hypertension and right ventricular hypertrophy during chronic hypoxia in Ddah1 knockout mice. Front. Physiol..

[44] Aggarwal S, Gross CM, Kumar S, Dimitropoulou C, Sharma S, Gorshkov BA, Sridhar S, Lu Q, Bogatcheva NV, Jezierska-Drutel AJ, Lucas R, Verin AD, Catravas JD, Black SM (2014). Dimethylarginine dimethylaminohydrolase II overexpression attenuates LPS-mediated lung leak in acute lung injury. Am. J. Respir. Cell Mol. Biol..

[45] Hannemann J, Böger R (2022). Dysregulation of the nitric Oxide/dimethylarginine pathway in hypoxic pulmonary vasoconstriction-molecular mechanisms and clinical significance. Front. Med. (Lausanne)..

[46] Wang D, Gill PS, Chabrashvili T, Onozato ML, Raggio J, Mendonca M, Dennehy K, Li M, Modlinger P, Leiper J, Vallance P, Adler O, Leone A, Tojo A, Welch WJ, Wilcox CS (2007). Isoform-specific regulation by N(G), N(G)-dimethylarginine dimethylaminohydrolase of rat serum asymmetric dimethylarginine and vascular endothelium-derived relaxing factor/NO. Circ. Res..

[47] Hoffmann M, Kleine-Weber H, Schroeder S, Krüger N, Herrler T, Erichsen S, Schiergens TS, Herrler G, Wu NH, Nitsche A, Müller MA, Drosten C, Pöhlmann S (2020). SARS-CoV-2 cell entry depends on ACE2 and TMPRSS2 and is blocked by a clinically proven protease inhibitor. Cell.

[48] Zhou P, Yang XL, Wang XG, Hu B, Zhang L, Zhang W, Si HR, Zhu Y, Li B, Huang CL, Chen HD, Chen J, Luo Y, Guo H, Jiang RD, Liu MQ, Chen Y, Shen XR, Wang X, Zheng XS, Zhao K, Chen QJ, Deng F, Liu LL, Yan B, Zhan FX, Wang YY, Xiao GF, Shi ZL (2020). A pneumonia outbreak associated with a new coronavirus of probable bat origin. Nature.

[49] Abou-Arab O, Huette P, Debouvries F, Dupont H, Jounieaux V, Mahjoub Y (2020). Inhaled nitric oxide for critically ill Covid-19 patients: A prospective study. Crit Care..

[50] Adusumilli NC, Zhang D, Friedman JM, Friedman AJ (2020). Harnessing nitric oxide for preventing, limiting and treating the severe pulmonary consequences of COVID-19. Nitric Oxide.

[51] Tavazzi G, Pozzi M, Mongodi S, Dammassa V, Romito G, Mojoli F (2020). Inhaled nitric oxide in patients admitted to intensive care unit with COVID-19 pneumonia. Crit. Care..

[52] Santamarina MG, Beddings I, Lomakin FM, Boisier Riscal D, Gutiérrez Claveria M, Vidal Marambio J, Retamal Báez N, Pavez Novoa C, Reyes Allende C, Ferreira Perey P, Gutiérrez Torres M, Villalobos Mazza C, Vergara Sagredo C, Ahumada Bermejo S, Labarca Mellado E, Barthel Munchmeyer E, Marchant Ramos S, Volpacchio M, Vega J (2022). Sildenafil for treating patients with COVID-19 and perfusion mismatch: a pilot randomized trial. Crit. Care..

[53] Fiorentino G, Coppola A, Izzo R, Annunziata A, Bernardo M, Lombardi A, Trimarco V, Santulli G, Trimarco B (2021). Effects of adding L-arginine orally to standard therapy in patients with COVID-19: A randomized, double-blind, placebo-controlled, parallel-group trial. Results of the first interim analysis. EClinicalMedicine..

