# Author Correction: The role of asymmetric dimethylarginine (ADMA) in COVID-19: association with respiratory failure and predictive role for outcome

**DOI:** 10.1038/s41598-023-37582-3

**Published:** 2023-06-27

**Authors:** Emanuela Sozio, Juliane Hannemann, Martina Fabris, Adriana Cifù, Andrea Ripoli, Francesco Sbrana, Demetrio Cescutti, Luigi Vetrugno, Stefano Fapranzi, Flavio Bassi, Massimo Sponza, Francesco Curcio, Carlo Tascini, Rainer Böger

**Affiliations:** 1 Infectious Diseases Clinic, Azienda Sanitaria Universitaria del Friuli Centrale (ASUFC), Udine, Italy; 2 Institute of Clinical Pharmacology and Toxicology, University Medical Center Hamburg-Eppendorf, Hamburg, Germany; 3 Institute DECIPHER, German-Chilean Institute for Research on Pulmonary Hypoxia and Its Health Sequelae, Hamburg, Germany; 4 Istituto di Patologia Clinica, Azienda Sanitaria Universitaria Friuli Centrale - Udine (ASUFC), Udine, Italy; 5 Department of Medical Area (DAME), University of Udine, Udine, Italy; 6 Bioengineering Department, Fondazione Toscana Gabriele Monasterio, Pisa, Italy; 7 Lipoapheresis Unit - Reference Center for Diagnosis and Treatment of Inherited Dyslipidemias, Fondazione Toscana “Gabriele Monasterio”, Via Moruzzi 1, 56124 Pisa, Italy; 8 Department of Medical, Oral and Biotechnological Sciences, University of Chieti-Pescara, Chieti, Italy; 9 Emergency Radiology Department - Azienda Sanitaria, Universitaria del Friuli Centrale (ASUFC), Udine, Italia; 10 Department of Anesthesia and Intensive Care Medicine, Azienda Sanitaria Universitaria Friuli Centrale - Udine (ASUFC), Udine, Italy; 11 U.O. Malattie Infettive, Azienda Sanitaria Universitaria Integrata di Udine, Via Pozzuolo, 330, 33100 Udine, Italy

Correction to: *Scientific Reports* https://doi.org/10.1038/s41598-023-36954-z, published online 17 June 2023

The original version of this Article contained an error in the spelling of the author Rainer Böger which was incorrectly given as Rainer Böeger.

The original Article has been corrected.

