# Supplementary material for: The role of asymmetric dimethylarginine (ADMA) in COVID-19: association with respiratory failure and predictive role for outcome
Source: Sci Rep. 2023 Jun 17;13:9811. doi: 10.1038/s41598-023-36954-z (PMC10276836; doi:10.1038/s41598-023-36954-z)
Supplement: Supplementary file 1 — Supplementary Tables. [file 41598_2023_36954_MOESM1_ESM.docx]

**Supplementary Table 1.** Clinical characteristics and laboratory findings of survivors *vs.* deceased patients.

|  | **Overall**  **(n = 90)** | **Survivors**  **(n = 80)** | **Deceased**  **(n = 10)** | **p** |
| --- | --- | --- | --- | --- |
| Female | 27 (30%) | 24 (30%) | 3 (27%) | 1.000 |
| Age (years) | 63 ± 12 | 63 ± 12 | 68 ± 5 | 0.005 |
| Cardiovascular disease | 17 (19%) | 15 (19%) | 2 (20%) | 1.000 |
| Diabetes | 12 (13%) | 10 (12%) | 2 (20%) | 0.869 |
| Charlson comorbidity index | 2 [1 - 4] | 2 [1 - 4] | 3 [2 - 4] | 0.197 |
| SOFA score | 3 [1 - 3] | 2[1-3] | 3 [3-5] | 0.024 |
| COVID-19 WHO Severity Classification | 3.7 ± 1.0 | 3.6+/- 0.9 | 4.6+/-0.7 | 0.002 |
| Lenght of hospital stay (days) | 11 [7 - 19] | 10 [7-16] | 17 [13-21] | 0.084 |
| ICU admission | 37 (41%) | 27 (34%) | 10 (100%) | <0.001 |
| IMV | 30 (33%) | 20 (25%) | 10 (100%) | <0.001 |
| PaO_2_/FiO_2_ ratio | 240 ± 108 | 248.56 +/- 109.23 | 167.2 +/- 84.56 | 0.026 |
| White blood cell (/mmc) | 6.72 ± 3.16 | 6.42 ± 2.98 | 8.92 ± 3.71 | 0.053 |
| Lymphocytes (/mmc) | 0.72 [0.50 - 1.01] | 0.75 [0.60 - 1.02] | 0.56 [0.29 - 0.75] | 0.097 |
| LDH (U/L) | 583 [443 - 814] | 578 [430 - 761] | 875 [622 - 1024] | 0.024 |
| C reactive protein (mg/dl) | 58.7 [25.7 - 104.3] | 53.0 [23.6 – 95.4] | 103.9 [78.0 - 176.1] | 0.007 |
| MR-proADM (nMol/L) | 0.84 [0.68 - 1.27] | 0.83 [0.68 - 1.23] | 0.97 [0.79 - 1.67] | 0.301 |
| IL-6 (pg/ml) | 29.0 [12.2 - 65.5] | 28.0 [12.2 – 65.0] | 37.0 [15.5 – 85.0] | 0.520 |
| ADMA (µmol/L) | 0.62 ± 0.20 | 0.59 ± 0.18 | 0.84 ± 0.20 | 0.002 |
| L-arginine (µmol/L) | 145.78 ± 54.53 | 146.89 ± 57.14 | 137.564 ± 29.27 | 0.404 |
| L-arginine / ADMA | 252.28 ± 120.88 | 263.95 ± 123.95 | 166.34 ± 28.70 | <0.001 |
| SDMA (µmol/L) | 0.57 [0.45 - 0.70] | 0.57 [0.45 - 0.69] | 0.53 [0.45 - 0.71] | 0.914 |
| Chest CT pulmonary vasodilation | 52 (56%) | 47 (58%) | 5 (46%) | 0.910 |

**Supplementary Table 2**. Clinical characteristics and laboratory findings of survivors vs. deceased patients that received IMV.

|  | **Overall patients**  **Received IMV**  **(n = 30)** | **Survivors (n = 20)** | **Deceased (n = 10)** | **p** |
| --- | --- | --- | --- | --- |
| Female | 7 (22%) | 4 (20%) | 3 (30%) | 542 |
| Age (years) | 65 ± 11 | 64 ± 9 | 69 ± 5 | 0.053 |
| Cardiovascular disease | 5 (17%) | 4 (20%) | 1(10%) | 0.488 |
| Diabetes | 4 (13%) | 2 (10%) | 2 (20%) | 0.448 |
| Charlson comorbidity index | 3 [2 - 3] | 2 [2 – 3] | 3 [2 – 4] | 0.313 |
| SOFA score | 3 [3 - 3] | 3 [3 - 4] | 3 [3 - 4.5] | 1.000 |
| COVID-19 WHO Severity Classification | 4.5 +/- 0.4 | 4.4 +/- 0.6 | 4.6 +/- 0.7 | 0.584 |
| Length of hospital stay (days) | 22 [14 - 33] | 25 [19 - 37] | 17 [13 - 21] | 0.055 |
| PaO_2_/FiO_2_ ratio | 192 +/- 89 | 204.15 +/- 90.68 | 167.2 +/- 84 | 0.292 |
| White blood cell (/mmc) | 8.87 ± 2.22 | 9.05 ± 3.39 | 8.92 ± 3.71 | 0.925 |
| Lymphocytes (/mmc) | 0.63 [0.30- 0.98] | 0.67 [0.36 – 0.96] | 0.56 [0.29 – 0.75] | 0.513 |
| LDH (U/L) | 813 [576 - 766] | 816 [578 – 1011] | 875 [622 – 1024] | 0.845 |
| C reactive protein (mg/dl) | 101.8 [58.5 – 161.1] | 90.7 [47.8 – 132.5] | 103.9 [78.0 – 176.1] | 0.411 |
| MR-proADM (nMol/L) | 1.21 [0.82 - 1.74] | 1.23 [0.84 – 1.72] | 0.97 [0.79 – 1.67] | 0.620 |
| IL-6 (pg/ml) | 29.5 [15.5 – 73.0] | 26.0 [15.0 – 58.0] | 37.0 [15.5 – 85.0] | 0.937 |
| ADMA (µmol/L) | 0.79 ± 0.21 | 0.75 ± 0.21 | 0.84 ± 0.20 | 0.262 |
| L-arginine (µmol/L) | 148.91 ± 44.46 | 151.17 ± 50.69 | 137.64 ± 29.27 | 0.347 |
| L-arginine / ADMA | 195.09 ± 62.97 | 205.91 ± 70.42 | 166.34 ± 28.70 | 0.033 |
| SDMA (µmol/L) | 0.58 [0.45 - 0.71] | 0.58 [0.45 – 0.71] | 0.53 [0.45 – 0.71] | 0.938 |
| Chest CT pulmonary vasodilation | 15 (50%) | 10 (50%) | 5 (50%) | 1.000 |
